# MRI Staging in Locally Advanced Vulvar Cancer: From Anatomy to Clinico-Radiological Findings. A Multidisciplinary VulCan Team Point of View

**DOI:** 10.3390/jpm11111219

**Published:** 2021-11-18

**Authors:** Benedetta Gui, Salvatore Persiani, Maura Miccò, Vincenza Pignatelli, Elena Rodolfino, Giacomo Avesani, Valerio Di Paola, Camilla Panico, Luca Russo, Simona Maria Fragomeni, Giorgia Garganese, Luca Tagliaferri, Giovanni Scambia, Riccardo Manfredi

**Affiliations:** 1 Area Diagnostica per Immagini, Dipartimento Diagnostica per Immagini, Radioterapia Oncologica ed Ematologia, Fondazione Policlinico Universitario A. Gemelli IRCCS, 00168 Rome, Italy; maura.micco@policlinicogemelli.it (M.M.); elena.rodolfino@policlinicogemelli.it (E.R.); giacomo.avesani@policlinicogemelli.it (G.A.); valerio.dipaola@policlinicogemelli.it (V.D.P.); camilla.panico@guest.policlinicogemelli.it (C.P.); lucarusso.md@gmail.com (L.R.); riccardo.manfredi@policlinicogemelli.it (R.M.); 2 Dipartimento Universitario di Scienze Radiologiche ed Ematologiche, Università Cattolica del Sacro Cuore, 00168 Rome, Italy; salvatorepersiani@gmail.com (S.P.); vincenza.pignatelli01@icatt.it (V.P.); 3 UOC Ginecologia Oncologica, Dipartimento per la Salute della Donna, del Bambino e di Sanità Pubblica, Fondazione Policlinico Universitario A. Gemelli IRCCS, 00168 Rome, Italy; simona.fragomeni@policlinicogemelli.it (S.M.F.); giovanni.scambia@policlinicogemelli.it (G.S.); 4 Gynecology and Breast Care Center, Mater Olbia Hospital, 07026 Olbia, Italy; giorgia.garganese@materolbia.com; 5 Dipartimento di Scienze della Vita e Sanità Pubblica, Università Cattolica del Sacro Cuore, 00168 Rome, Italy; 6 UOC di Radioterapia Oncologica, Dipartimento Diagnostica per Immagini, Radioterapia Oncologica e Ematologia, Fondazione Policlinico Universitario A. Gemelli IRCCS, 00168 Rome, Italy; luca.tagliaferri@policlinicogemelli.it

**Keywords:** vulvar anatomy, vulvar cancer, MRI, female pelvic MRI

## Abstract

MR imaging provides excellent spatial and contrast resolution to stage locally advanced vulvar cancer (LAVC) for tumor and nodal evaluation in order to facilitate the planning of treatment. Although there are no standard indications for how to estimate the clinical stage of International Federation of Gynecology and Obstetrics at diagnosis, MR imaging can depict the tumor and its extension to the vulvar region and adjacent organs, such as the vagina, urethra, and anus. Optimizing the MR imaging protocol and technique is fundamental for correct staging. The aim of this overview was to focus on the role of MR imaging in LAVC staging. We define vulvar anatomy and corresponding MR imaging findings, MR imaging protocol, and technique. Moreover, we describe the MR imaging findings of LAVC with example cases stage by stage. Key imaging findings based on signal intensity, diffusion restriction, and enhancement are portrayed to correctly identify and stage vulvar cancer. A structured report for LAVC staging is reported in order to give all necessary information to the clinicians and to facilitate MR imaging comprehension.

## 1. Introduction

Primary vulvar cancer is a rare tumor accounting for approximately 4% of all gynecologic malignancies [1]. Squamous cell carcinoma (SCC) is the most usual type (>85% of all vulvar cancers) [2]. Melanoma, extramammary Paget’s disease, Bartholin gland cancer, verrucous carcinoma, basal cell carcinoma, and sarcoma are other rare vulvar malignancies [1,3,4]. Currently, there is no screening procedure for vulvar cancer. There are two type of SCC based on association with human papilloma virus (HPV). HPV-positive cancers mainly arise in women younger than 60 years and are correlated with vulvar intraepithelial neoplasia (VIN), have a tendency to be multifocal and multicentric, and may be combined with analogous lesions of the cervix and vagina [5,6,7]. HPV-negative cancers mostly arise in old women (>60 years) and are correlated with vulvar inflammation and lichen sclerosus. Typically, these lesions are unifocal and well differentiated due to formation of exuberant keratin [5,6,7]. Vaccination may reduce the burden of HPV related cancer in the future [8].

Generally, vulvar cancer is most common in postmenopausal women, and the median age at diagnosis is 68 years. Diagnosis is made by physical examination and biopsy [1]. The most important prognostic factors include size of the primary tumor and the regional lymph node status, which is also a determinant for treatment [1,9,10]. Staging for vulvar cancer follows the International Federation of Gynecology and Obstetrics (FIGO) and American Joint Committee on Cancer (AJCC) staging systems, which closely align [11]. (Table 1). 

Both staging systems are mainly based on the pathological report, but in a clinical routine it is necessary to achieve an adequate estimation of the disease to better tailor the treatment plan. In this field, imaging performs a crucial role and MRI is the imaging modality of choice, as was recently published on the vulvar cancer staging guidelines by the Female Pelvic Imaging Working Group of the European Society of Urogenital Radiology (ESUR) [11].

In fact, MRI shows a great potential in the study of the tumor and the surrounding soft tissues aside from the evaluation of locoregionally involved nodes. Otherwise, a PET/CT or CT scan allows one to identify distant and nodal metastases. 

Vulvar cancer is staged pathologically by an evaluation of tumor size and the depth of invasion determined by (1) physical examination; (2) biopsy/surgical pathology; and (3) lymph node status assessed by physical examination, imaging, or surgical removal via lymphadenectomy or sentinel lymph node biopsy [1,5,12,13]. Some authors report that most vulvar cancers involve the labia majora and minora (70%) with other sites involved in the remaining cases [10,12,14]. Vulvar cancer generally extends slowly and tends to be locally infiltrative prior to invading the local lymph nodes in the groin. Regional extension may involve the vagina, urethra, and anus, but rarely the bladder and bone. In more advanced cases, the pelvic nodes can be involved [10,15]. Metastatic diffusion outside the pelvis is unusual in vulvar cancer aside from malignant melanoma and rare sarcomas [10].

Previous studies show that pelvic MRI and PET/CT help over clinical assessment in the pre-treatment management of patients with vulvar cancer [16,17,18]. MRI is the preferred technique for the assessment of locally advanced vulvar cancer (LAVC) and its extension to the adjacent structures. Kataoka et al. reported an accuracy of 85% for staging vulvar cancer with enhanced MRI [19]. Moreover, some studies have evaluated the role of MRI in detecting lymph node metastasis, reporting large varying sensitivities and specificities ranging from 40% to 89% and 81% to 100%, respectively [12].

LAVC includes unresectable by non-visceral sparing primary surgery large T2 and T3 tumors, as outlined by the National Comprehensive Cancer Network [1]. The expression “locally advanced vulvar carcinoma” has been related to different clinical presentations including bulky primary tumors spreading beyond the vulva or presenting with large positive groin nodes; tumors either near or involving the adjacent organs, such as the vagina, urethra, bladder, anus and/or rectum or pelvic bones; and primary tumors that cannot be locally managed with a radical vulvar resection [20,21].

The treatment of vulvar cancer should be personalized, including tailored primary tumor resection and lymph nodes evaluation and/or primary chemoradiation therapy or exclusive chemo-radiation based on an individual patient’s characteristics [1]. In this setting, patients with vulvar cancer should be referred to a dedicated cancer center as diagnosis and management should be multidisciplinary with a dedicated team, including a gynecologist, radiation oncologist, radiologist, nuclear medicine physician, medical oncologist, pathologist, and plastic surgeon [22,23,24,25]. 

This is a narrative review with the specific aim of integrating the anatomy and MRI findings of the vulvo-perineal region in both normal and cancer related settings. Moreover, this paper focuses on the integration between the needs of the clinician and the MRI findings of LAVC in order to plan combined treatments specific to each patient.

The close collaboration between radiologist, radiation oncologist, and gynecologic oncologist allows one to reach the maximum diagnostic accuracy and to improve the performance of the treatment.

## 2. Materials and Methods

A review of the published literature was conducted by searching several electronic databases from 2000 to 2021, including Medline/Pubmed, Scopus, Embase, and the Cochrane library. The search of these online databases was conducted by three expert investigators (two radiologists and one resident in radiology). The search included combined key terms and exploded Medical Subject Headings (MeSH). 

**Figure 1:**
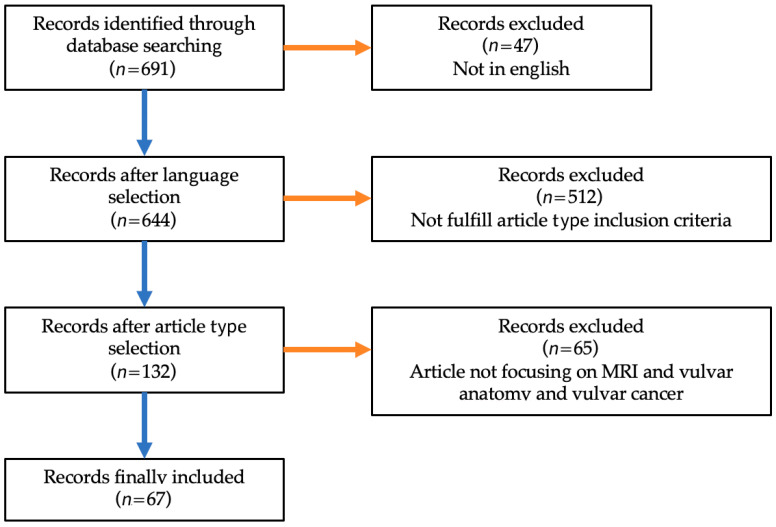
Flow diagram for studies’ selection.

The terms we focused on were the following: “vulvar”, “vulva”, and “female perineum”, and “mri”, “magnetic resonance imaging”, and “magnetic resonance”. The inclusion criteria were: english-language human-based studies, in more details reviews, longitudinal and retrospective in vivo studies and guidelines. Exclusion criteria were: (1) ex vivo studies and (2) case report (Figure 1). Table 2 provides a summary of the most relevant literature concerning the role of MRI in staging vulvar cancer, including guidelines, review, and retrospective studies.

## 3. Anatomy and MRI Findings

**Figure 2:**
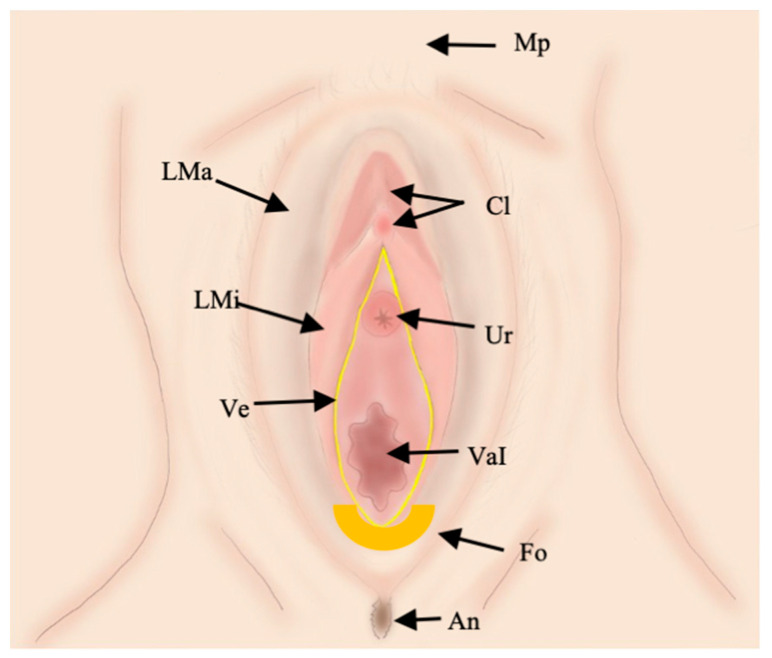
Illustration of vulvar anatomy. The superficial structures of the vulva are shown. Mons pubis (Mp), labia majora (LMa), labia minora (LMi), clitoral glans and prepuce (Cl), vestibule (Ve), including urethral meatus (Ur) and vaginal introitus (VaI). Posteriorly, there are the fourchette (Fo) and the anus (An).

The vulva is a triangle-shaped structure of the external female genitalia bounded externally by the skin and profoundly by the urogenital diaphragm, anteriorly by the symphysis pubis, posteriorly by the anal sphincter, and laterally by the ischial tuberosity [5,7,12,15]. The vulva is formed by the following structures: mons pubis, labia majora and labia minora, clitoris, the vestibular bulbs, the bulbo-spongiosus, the ischio-cavernosus muscles, and the vestibule, containing the external urethral meatus and vaginal introitus [5,7,12,15] (Figure 2). 

### 3.1. Mons Pubis, Labia Majora and Minora

**Figure 3:**
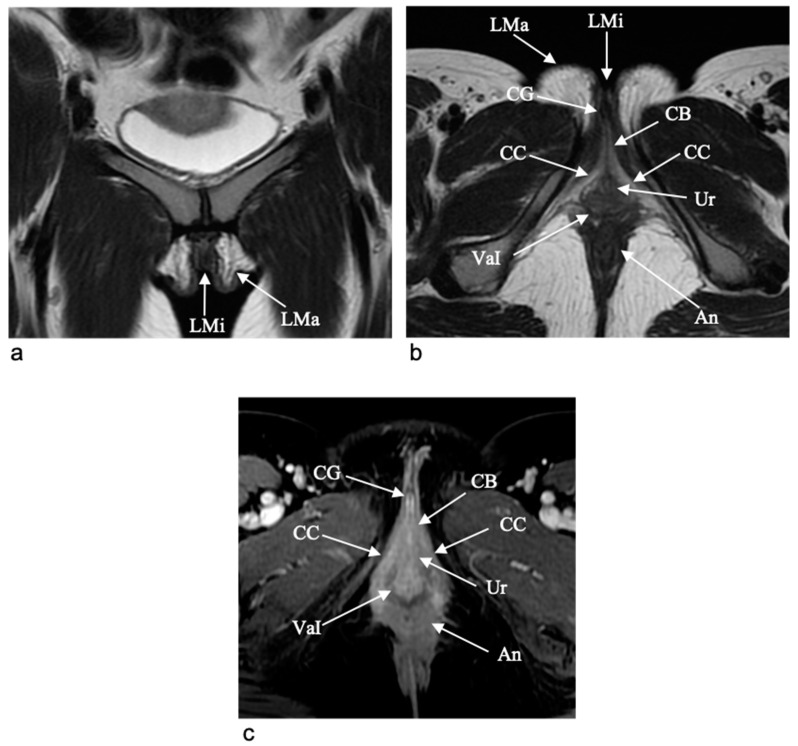
MR images of vulvar structures. On coronal (**a**) and axial (**b**) T2-W MR images the labia majora (LMa) show high signal intensity and the labia minora (LMi) show low signal intensity. On the axial T2W MR image (**b**) the clitoral anatomy is well evaluated with identification of the different components, such as the crura (CC), the body (CB) and the glans (CG), which show higher signal intensity than muscle and the contiguous structures. Axial contrast-enhanced T1-W MR image (**c**) shows a marked contrast enhancement of the clitoris; the midline septum of glans is also evident (CG). Within the labia minora there is the vestibule including the urethral meatus (Ur) and vaginal introitus (VaI). Posteriorly there is the anus (An).

The mons pubis consists of fatty tissue covering the symphysis pubis and extending deeply into two separate large anterior and lateral folds of skin known as the labia majora [5,7,12,15,27]. The labia majora are thicker in front joining and forming the anterior commissure, whereas they remain parallel each other without really joining posteriorly, only forming with the connecting skin as the posterior commissure, which represents the posterior boundary of the vulval orifice. The region called “fourchette” is the anterior edge of the perineum between the vulva and the anus. The mons pubis and the labia majora are hyperintense on both T1-weighted (T1-WI) and T2-weighted images (T2-WI) due to their fatty tissue content (Figure 3). 

The labia minora are two thin folds located medially to the labia majora and fused anteriorly at the level of the glans of the clitoris that terminates between the orifice of the vagina and the labia majora [5,7,12,15]. Labia minor show hypointense signal on T2-weighted images (Figure 3). 

### 3.2. Clitoris 

The clitoris is a pyramid structure formed by erectile parts such as crura, bodies and bulbs, and non-erectile tissue such as the glans [27]. The right and left clitoral crura are parallel and medial to the ischio-pubic rami and join into the clitoral bodies [5,7,12,15,27]. The clitoris bodies are composed of two corpora cavernosa with a “boomerang-like” form and arise posteriorly as the crura and join anteriorly as a single body; the most caudal portion is adjacent to the glans [27]. The vestibular bulbs are paramedian erectile tissue, parallel to the crura, which enclose the urethra and vagina anterolaterally and convene anteriorly at the commissure, ventral to the urethra and close to the body and glans. The glans is the round small free extremity, partially external and projecting into the mons pubic fat [27]. 

The ischiocavernosus muscles envelope the crura and join the ischiopubic rami, supporting clitoral erection with the bulbospongiosus muscles. There is no difference in clitoral morphology between premenopausal and postmenopausal women [27]. The clitoris commonly shows higher signal intensity on T2-weighted images than muscle and contiguous structures and marked contrast enhancement due to their great vascularity (the clitoral bulb enhances slightly less than the remainder of the clitoris) (Figure 3) [7]. The gland usually shows a midline septum [27]. The clitoris shows a curved configuration and is detectable on some images. Clitoral anatomy is best viewed in the axial plane with sagittal and coronal planes supplying more details, remembering that each plane provides a different representation of the structure. Sagittal planes show the angled “boomerang-shaped” clitoral body and glans at the undersurface of the pubic symphysis. Coronal plane shows the two corpora joining into a single body and ending in the glans as well as delineates the labia minora and majora [27]. 

### 3.3. Vestibule

The vestibule of the vulva is the area within the labia minora containing the vaginal introitus and the urethral meatus (Figure 3). The Bartholin glands are located posterolateral and bilaterally in the introitus and secrete lubricants into the vestibule [5,7,12,15]. 

**Figure 4:**
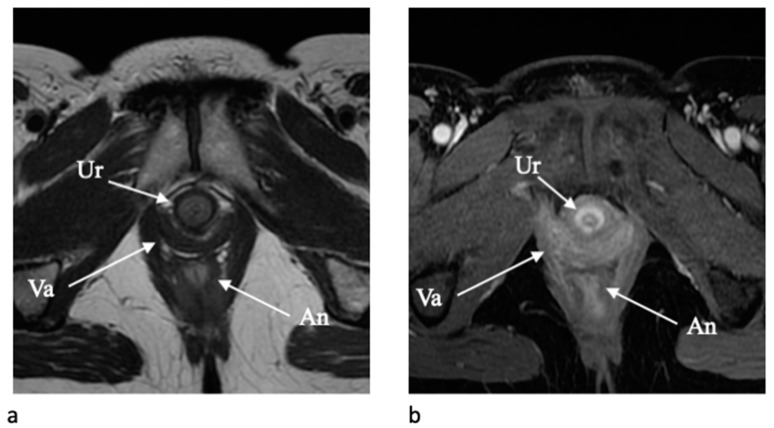
MR images of the urethra. Axial T2-W MR image (**a**) shows a typical target-like appearance of urethra (Ur), with an outer low-signal-intensity ring of external skeletal muscle of the urethral sphincter and a high signal intensity inner layer of smooth muscle and submucosa. Axial contrast-enhanced T1-W MR image (**b**) shows the corresponding aspect of the urethra (Ur) after contrast administration with evidence of an outer ring of tissue with less enhancement, representing the external skeletal muscle of the urethral sphincter, surrounding a circular band of higher enhancement of smooth muscle and submucosa. Posteriorly there are the vagina (Va) and the anus (An).

The urethra is clearly depicted on axial T2-weighted and contrast-enhanced T1-weighted images due to a typical target-like appearance with an outer low-signal-intensity layer of external skeletal muscle of the urethral sphincter and a high signal intensity inner layer of smooth muscle and submucosa [7] (Figure 4). The vagina is similarly well depicted on axial T2-WI and axial post-contrast T1-WI, and the shape resemble an “H” form. The vaginal wall includes the mucosal layer (hyperintense on T2-WI with high vascularization) and the submucosal and muscolaris layers (hypointense on T2-WI). The paravaginal fatty tissue presents a high signal intensity on T2-WI [5,7].

### 3.4. Vascularization

The vulva is vascularized via branches of the external and internal pudendal arteries. The venous drainage is mainly via the (external and internal) pudendal veins, perineal vein, and deep posterior vein of the clitoris [7,31].

### 3.5. Lymphatic Drainage

The lymphatic drainage of the vulva happens mainly through the superficial inguinal lymph nodes [5,32] located just below the skin and above the fascia lata [33]. From superficial inguinal nodes the metastasis spread to the deep inguinal lymph nodes, located below the fascia lata, along the femoral artery and vein, and subsequently to the external iliac lymph nodes. The cloquet node, the lowest of the external iliac lymph nodes, is at the entrance of the femoral canal and is an important indicator of metastatic spread to the pelvic nodes [10]. The lymphatic drainage of the vulva follows a rich network of lymphatic anastomoses, which continue over the midline. For this reason, the pattern of lymph node metastasis is not restricted to one side and a lesion near the midline can drain to one or both groins [5,32]. Moreover, the clitoris can drain directly to the deep inguinal or external iliac lymph nodes through the hypogastric route [32,34]. The inguinal and femoral lymph nodes are considered locoregional, whereas the involvement of the pelvic lymph nodes is rare and is considered metastatic disease [5,12,32].

**Figure 5:**
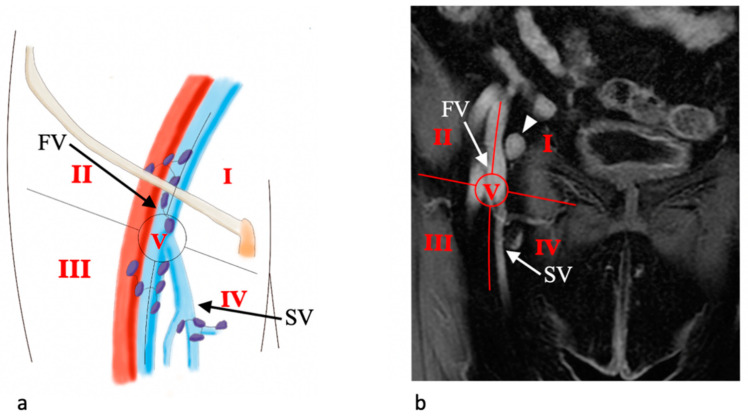
Daseler classification for inguinal lymph nodes. Illustration (**a**) and coronal contrast-enhanced T1-W MR image (**b**) show the sapheno-femoral junction (circle) with the corresponding defined five regions: medial superior region (I), medial inferior region (IV), central region (V), lateral superior region (II), and lateral inferior region (IV). The saphena vein (SV) and femoral vein (FV) can be clearly distinguished. An enlarged inguinal lymph node (arrowhead) is detected in region I in (**b**). Illustration (**a**) is modified from Protzel et al. [33].

In our institution, we use the Daseler classification for inguinal lymph nodes anatomic localization for vulvar cancer. Daseler et al. divided the inguinal region into five zones marked by four quadrants obtained by drawing vertical and horizontal lines over the sapheno-femoral junction, and one zone directly overlying this junction [35]. As a result, we acknowledge one medial superior region, one medial inferior region, one central region, one lateral superior region, and one lateral inferior region (Figure 5). 

This anatomic classification is important because, looking at the distribution of sentinel lymph nodes (SLNs), it has been shown that lymphatic drainage occurs mainly to the medial regions of the groin as well as the distribution of metastatic SLNs [36]. Moreover, drainage to the lateral inferior region of the groin is only incidental. 

## 4. MRI Protocol

According to ESUR guidelines, the minimal recommended magnet field strength for staging is 1.5 T [11]. Patient preparation and imaging techniques are essential to achieve an optimal examination. Fasting for 4–6 h before the examination, anti-peristaltic agent administration, and bladder voiding are required to limit bowel peristalsis. Vaginal distention with ultrasound gel should be useful for the delineation of small vulvar tumors and/or vaginal infiltration [5,7,11,12]. The patient should be imaged supine, with a phased array pelvic coil or an eight-channel cardiac coil [14]. The standard MRI protocol used in our department for the evaluation of vulvar lesions is summarized in Table 3. It includes axial T1-wieghted images (T1WI) and T2-weighted images (T2WI) with a large field of view (FOV) for an overview of the whole pelvis, allowing detection of lymphadenopathy and pelvic bone metastasis [5,7,12,31]. Sagittal T2WI FSE, oblique axial (perpendicular to the long axis of the urethra), and coronal oblique (parallel to the axis of the urethra) high resolution T2WI provide anatomic detail and tumor delineation [5,7,12,31]. Some authors suggest the use of T2WI with fat suppression (FS) as they provide a better delineation of the tumor, given that the perineal region is rich in fat. Oblique axial diffusion-weighted imaging (DWI) with a high b-value (high b = 1000) (with calculated apparent diffusion coefficient [ADC] map) on the same angle of oblique axial T2WI is also acquired for a better delineation of the primary tumor.

Three-dimensional spoiled gradient-pulse fat-saturated T1WI (3D T1WI FS) imaging of the pelvis on the axial or axial oblique plane (perpendicular to the long axis of the urethra) are performed pre- and post-contrast administration, after 18 s of delay and at 15 s intervals for 3 scans to better obtain arterial portal and equilibrium phases. Coronal or oblique coronal plane (parallel to the long axis of the urethra), and eventually sagittal plane, post-contrast enhanced images are subsequently performed. Dynamic contrast-enhanced MRI helps in the evaluation of small tumors and of the involvement of the urethra, anus, and vagina. Multiplanar imaging is essential in evaluating the anatomy and extension of the tumor to the adjacent structures.

The evaluation of the upper abdomen to assess the kidney and lymph nodes should be performed and include axial T2WI (and eventually DWI) from the symphysis to the renal hila.

## 5. MR Imaging Impact Stage by Stage

**Figure 6:**
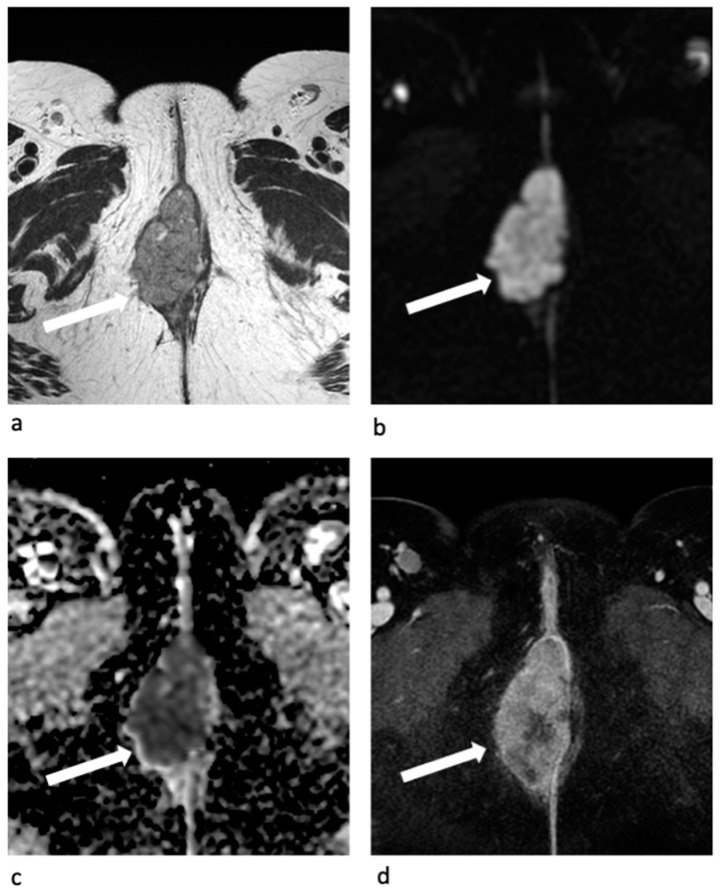
MR images of vulvar tumor. The tumor (arrow) shows intermediate hyperintense signal intensity on axial T2-W MR image («evil grey») (**a**) and hyperintense signal intensity on DW-MR image (**b**) with restricted diffusion on the corresponding ADC map (**c**) due to the high cellularity. On the axial contrast-enhanced T1-W MR image (**d**) acquired during the arterial phase the tumor shows early and conspicuous enhancement.

Vulvar tumors appear as a solid mass, usually hypo-isointense on T1-WI, and moderately hyperintense on T2-WI (“evil grey”) in comparison to the muscles. Tumors show diffusion restriction on DWI and early arterial enhancement on the 3D dynamic-enhancement sequences (Figure 6). MRI is usually requested to better define the size of the tumor and its anatomic extension to the adjacent structures for LAVC, including the superficial (labia majora, minora, clitoris) and the deeper ones (urethra, vagina, and anus) [5,7,12,14,31].

- *Stage II*

**Figure 7:**
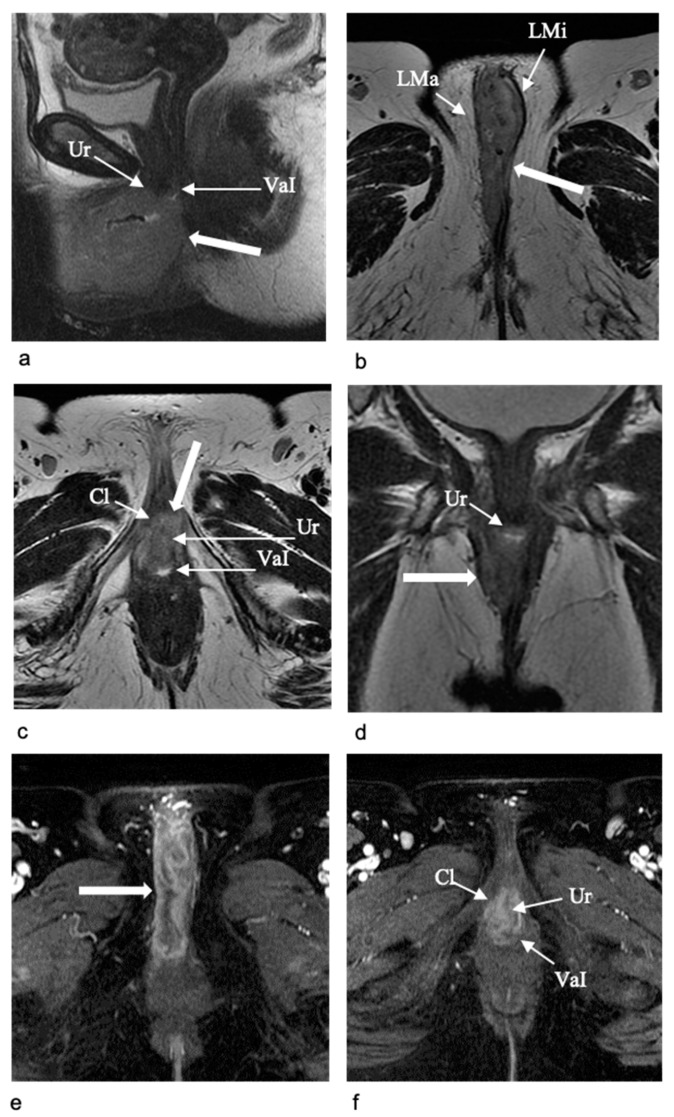
Stage II, in a 63-year-old woman with vulvar carcinoma. Sagittal (**a**) and axial T2-W MR images at the level of perineum (**b**) and of vestibule (**c**) show a bulky vulvar mass (arrow) involving the labia majora (LMa), labia minora (LMi), and clitoris (Cl), extending posteriorly to the urethral meatus (Ur) and vaginal introitus (VaI). On the coronal T2-W MR image (**d**) the involvement of the urethral meatus is also evident (Ur) by the tumor. Axial contrast-enhanced T1-W MR images at the level of perineum (**e**) and of vestibule (**f**) show the intense enhancement of the tumor and its extension to the clitoris (Cl), the urethral meatus (Ur) and the vaginal introitus (VaI) posteriorly.

In Stage II, a tumor of any size extends to the adjacent structures, including the distal third of the urethra, vagina, or anus, and no positive nodes. Clinical evaluation of urethral infiltration could be difficult, especially if the lesion is in contact with the urethral meatus but there are no certain findings of parietal infiltration. In this case, MRI identification of the interruption of the target aspect of the urethra on both T2WI and post-contrast T1WI is indicative of parietal infiltration [11,12,19] (Figure 7). On the other hand, if the lesion is adjacent to the urethra but the normal MRI signal characteristic are preserved on both T2-and post-contrast T1WI, parietal infiltration is unlikely. However, the surgeons need to know the closed extension between the tumor and the urethral meatus. Clinical evaluation of vaginal infiltration could also be difficult in case of a large tumor and/or vaginal stenosis. MRI disruption of the low signal intensity on T2WI of the vaginal wall, and of the parietal layers on post-contrast T1WI, are indicative of wall infiltration [11,12,19]. In the case of a posterior extension of the tumor, anal sphincter infiltration should be excluded, especially if clinical evaluation is indicative of a fixed tumor with fatty plane obliteration. As for vaginal infiltration, the disruption of the low signal intensity on T2WI of the anal sphincter, and of the muscular layers on post-contrast T1WI, are indicative of wall involvement. In doubtful cases, endo-anal sonography could be useful for a better evaluation of the muscle layers. Moreover, DWI sequences are extremely useful to assess the extension of the tumor into the urethra, vagina, and anus, showing the continuation of the restricted signal of the tumor into the urethral and/or vaginal wall and anal sphincter. The careful evaluation of the DWI sequences with the corresponding T2WI ones, on the same plane, is mandatory to identify the tumor, recognize the anatomy, and asses the involved adjacent structures [7,14]. 

Tumor localization is determinant for the choice of the primary treatment. Lateral vulvar lesions (i.e., sited > 1–2 cm from the midline) should be treated with radical local resection or modified radical vulvectomy, both with an assessment of the ipsilateral inguinal lymph node. Midline vulvar lesions should be treated with radical local resection or modified radical vulvectomy and an assessment of both inguinal lymph nodes [1,12,37]. Inguinal lymph node evaluation may be achieved through sentinel node biopsy (for unifocal tumors < 4 cm in patients without clinical or radiological evidence of lymph node disease) or inguinofemoral lymph node dissection (for tumors ≥ 4 cm, in cases of multifocal disease). If the tumor extends to the distal urethra or vagina, resection with clear margins is an option. If the tumor extends to the anus, preoperative radiotherapy, with or without concurrent chemotherapy, may be contemplated to avoid colostomy [1,12].

- *Stage III*

**Figure 8:**
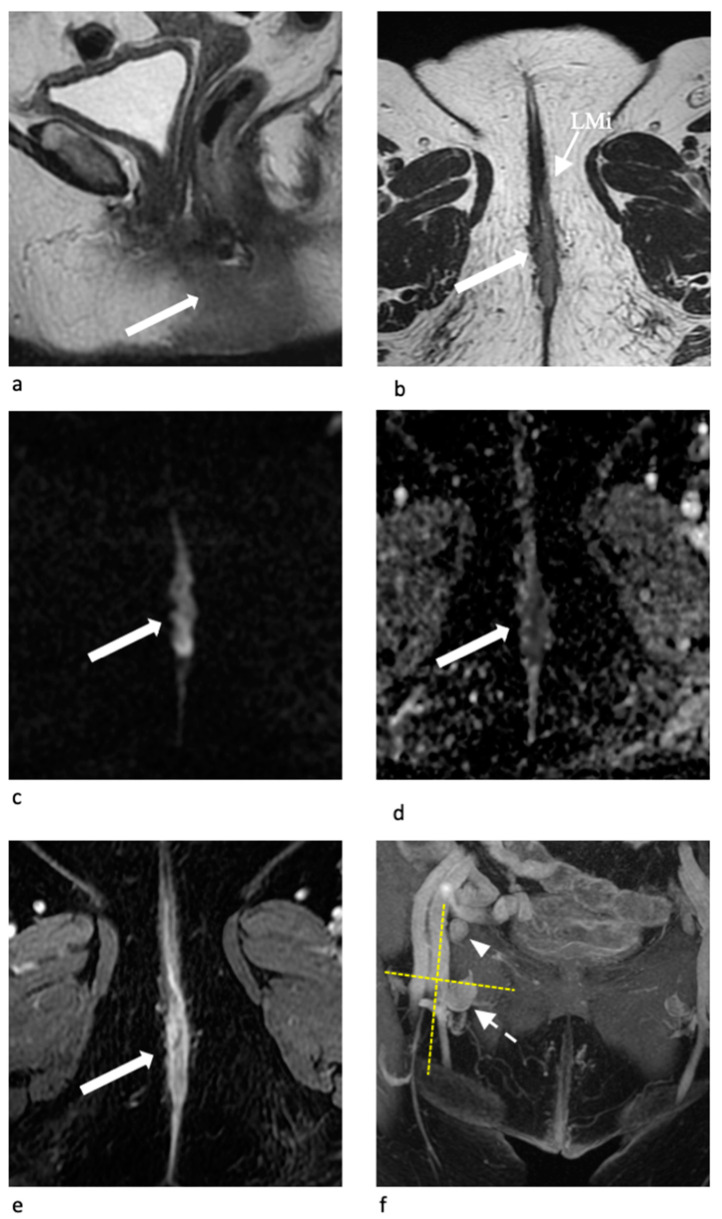
Stage III, in a 72-year-old woman with vulvar carcinoma. Sagittal (**a**) and axial T2-W (**b**) MR images show a midline vulvar mass (arrow) involving labia minora (LMi) and confined in the perineum without invasion of adjacent organs. Axial DW-MR image (**c**) and corresponding ADC map (**d**) show hyperintensity of the lesion with marked diffusion restriction. Axial contrast-enhanced T1-W MR images (**e**) show the early arterial enhancement of the tumor. On coronal maximum intensity projection (MIP) reformatted MR image (**f**) there are an enlarged right inguinal node in the IV Daseler level (dotted arrow) and a smaller one in the I level (arrowheads). Surgery was performed and stage III was confirmed.

The central point in Stage III is represented by the inguinal node involvement. In Stage IIIA, there is an involvement of one or two inguinal lymph nodes < 5 mm, or a single inguinal lymph node ≥ 5 mm. In Stage IIIB, there is an involvement of two or more inguinal lymph nodes ≥ 5 mm or three or more < 5 mm (Figure 8). In Stage IIIC, there are positive lymph nodes with extracapsular spread. Lymph nodes involvement is the primary prognostic factor in vulvar cancer and it is important for treatment planning and prognosis [1]. The primary critical determinant of overall survival for vulvar cancer is the presence of positive inguino-femoral lymph nodes, with a 5-year survival rate of approximately 50% [1,5]. The most accepted MRI characteristics suggestive of malignancy include (a) short axis diameter > 10 mm; (b) rounded morphology with short axis to long (S to L) axis ratio > 0.75; (c) necrotic changes within the lymph node; and (d) irregular contour and loss of fatty hilum [11].

To reduce a false positive rate, clinicians are suggested to schedule MRI at a time not too close to the performed vulvar biopsy [38]. 

Even if the staging FIGO system is based on the pathology report, the chance to estimate preoperatively the presence of inguinal lymph node metastases allows for a better planning of surgery. In general, it allows one to consider a patient for sentinel node biopsy in case of negative lymph nodes, or to decide for contralateral lymphadenectomy when a groin is grossly involved [1].

In case of suspicion, it may be useful to integrate the MRI images with an ultrasound in order to perform a needle aspiration or core biopsy for confirmation [39].

In case of midline lesions, mono or bilateral inguinofemoral dissection must be provided. After pathology report, the patient should undergo radiation therapy with eventual concurrent chemotherapy [1,12].

- *Stage IVA*

In Stage IVA (1), the tumor is fixed to and invades the pelvic bones or involves the upper urethral or vaginal mucosa, bladder, or rectum, or (2) results in fixed or ulcerated inguinofemoral lymph nodes. A clinical evaluation of the deep urethra, vagina, and rectum could be difficult and an MRI is necessary to better asses the tumor extension in case of suspected pelvic parietal involvement.

**Figure 9:**
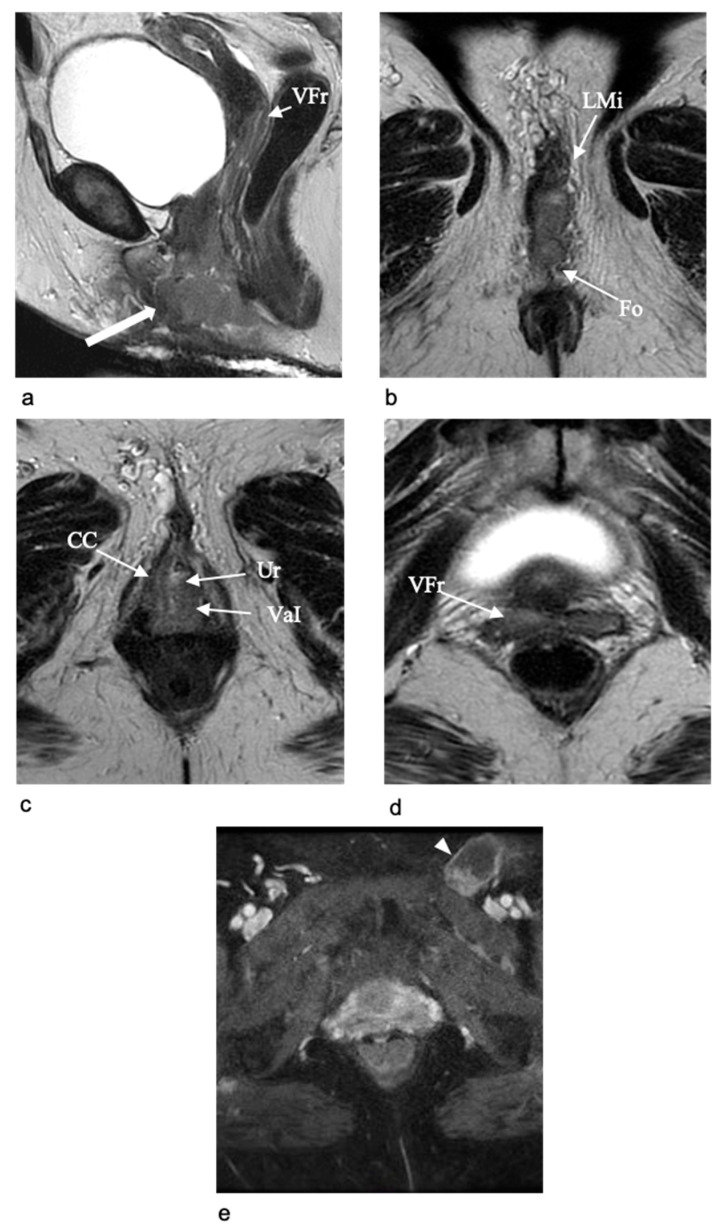
Stage IVA, in an 88-year-old woman with vulvar carcinoma. Sagittal (**a**) and axial T2-W MR images at the level of perineum (**b**), of vestibule (**c**) and of pubic symphysis (**d**) show a big vulvar mass (arrow) located mainly on the left vulvar side involving the labia minora (LMi) and the fourchette region (Fo) posteriorly. The tumor also infiltrates the clitoris crura (CC) and the vestibule, including the urethral meatus (Ur) and the vaginal introitus (VaI) and extends cranially to the upper vagina into the fornices (VFr). Axial contrast-enhanced T1-W MR image (**e**) shows an enlarged and necrotic left inguinal lymph node (arrowheads).

Urethral, vaginal, bladder, and rectal wall infiltration can be identified for the disruption by an intermediate signal tumor on T2WI and post contrast T1WI of the normal target appearance of the upper two thirds of the urethra, the low signal intensity of the upper two thirds of the vagina, or the normal high signal intensity of the bladder or rectal mucosa [5,12] (Figure 9). DWI sequences are also fundamental to assess tumor extension, as described above. Multiplanar evaluation is necessary to better identify and define the extension of the tumor to the adjacent organs. The sagittal plane is best option for evaluating the caudo-cranial extension of the tumor into the vagina, helping to identify the involvement of the upper portion of the vagina, including the vaginal fornices. The sagittal plane is also useful for the evaluation of the caudo-cranial urethral extension and of the bladder base. Rectal caudo-cranial involvement is also better appreciated on the sagittal plane with more easy distinction between anus and rectum, also on the coronal plane. Axial and coronal planes are both useful for the evaluation of the lateral tumor extension showing a better identification of the muscles and bones of the pelvis.

When, thanks to MRI staging, it is estimated that a clinical stage IVA is present due to local tissue infiltration or massive diffusion to the inguinal lymph nodes, patients who are naive to radiotherapy treatment are preferably referred to concomitant chemoradiation therapy. In such cases, the role of the MRI is fundamental at the beginning for the radiation treatment planning and after the end of the treatment for an adequate evaluation of the response.

- *Stage IVB*

**Figure 10:**
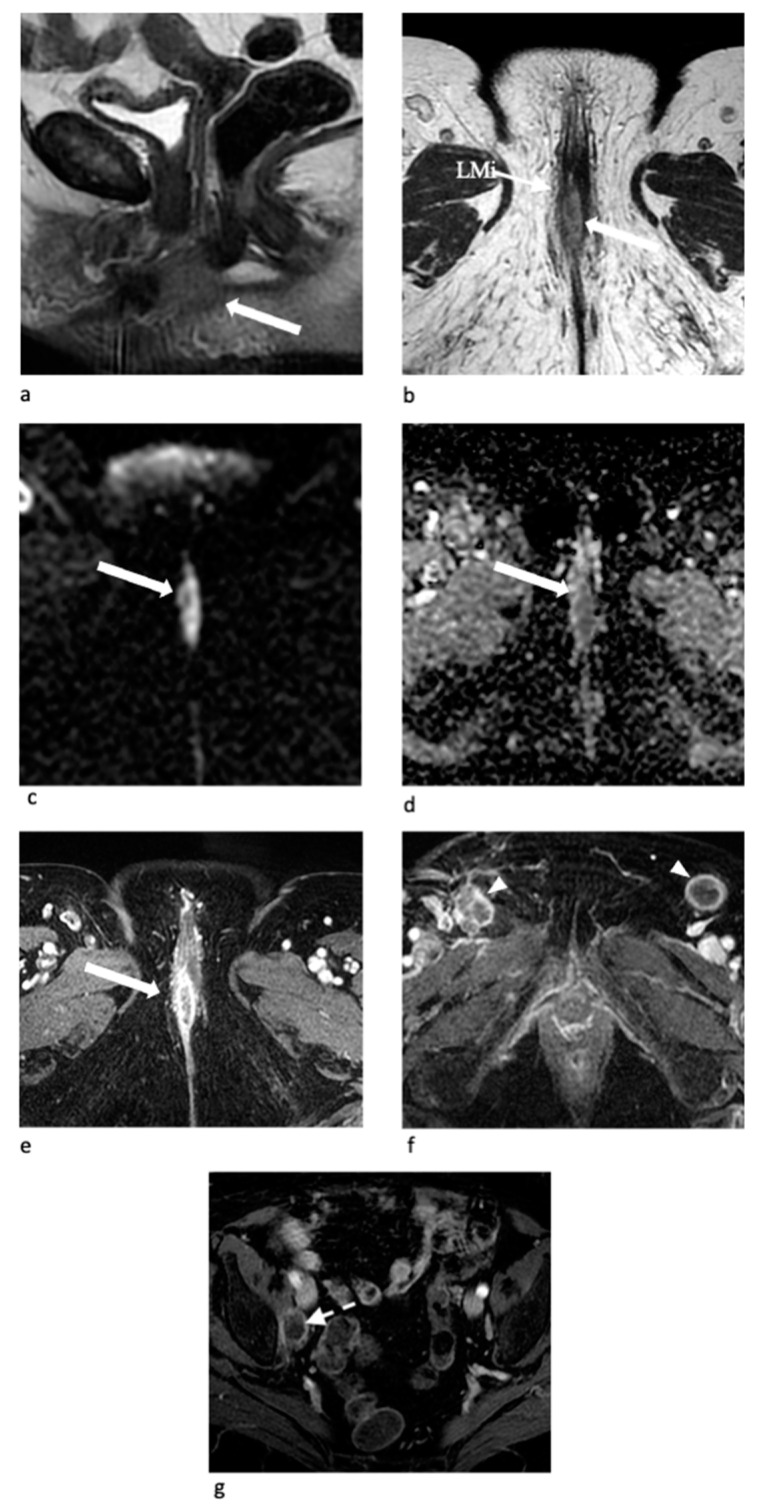
Stage IVB, in a 75-year-old woman with vulvar carcinoma. Sagittal (**a**) and axial (**b**) T2-W MR images show a midline vulvar mass (arrow) involving the right labium minus (LMi) with hyperintense signal intensity on axial DWI (**c**) and marked diffusion restriction on the corresponding ADC map (**d**). Axial contrast-enhanced T1-W MR images at the level of perineum (**e**) and of pubic symphysis (**f**) show early arterial enhancement of the tumor (arrow) and necrotic bilateral inguinal lymph nodes (arrowheads). Axial contrast-enhanced T1-W MR image on the pelvis also shows the presence of an enlarged and necrotic right obturator lymph node (dotted arrow) (**g**).

In stage IVB there is distant metastatic disease, including (1) pelvic lymph nodes and (2) distant organs (Figure 10).

As it is predictable, MRI is not able to provide a systemic staging of the disease, so, in case of suspicion it is necessary to integrate with other exams, such as 18F FDG PET or 18 F FDG PET/CT [40,41].

Chemoradiotherapy is the treatment preferred for all patients with pelvic stage IVB. Ultrasound guided fine-needle aspiration cytology/biopsy (FNAC/FNAB) should be performed in patients with imaging-positive lymph nodes [42] to confirm the diagnosis. 

The use of additional therapies depends on the response to chemoradiotherapy. After treatment, interventional radiotherapy boost (brachytherapy) or surgery should be considered if there is any evidence of residual tumor. In case of an unresectable residual tumor, additional local therapies, such as radiotherapy or electrochemotherapy, with or without systemic therapy, could be considered. In patients with distant metastatic disease, antiblastic systemic or local palliative treatment may be contemplated [1,12,43].

## 6. MRI Focused Report

A comprehensive report for staging LAVC requires the subsequent information: tumor dimension (maximum diameter); tumor location (lateral, midline, multifocal); clitoris involvement; tumor adjacent organs/structures extension (urethra and/or vagina with caudo-cranial extension specification: lower 1/3 or upper 2/3; urethral meatus; bladder; fourchette region; and anus/rectum); and lymph-nodes involvement: inguinal and/or pelvic and/or abdominal. Moreover, it is important to report additional findings regarding uterus, adnexa, kidneys, and pelvic bones [11].

## 7. Conclusions

Vulvar cancer is a rare malignancy. Multidisciplinary evaluation of patients with LAVC based on tumor board discussion assumes an important role in choosing the best correct and personalized treatment. In this setting, MR imaging provides an accurate anatomic evaluation of the vulvar region and is essential for the local staging of LAVC and lymph nodes evaluation. Optimal MRI technique allows for an accurate assessment of the deep extension of the tumor into adjacent structures in association with clinical examination. The vagina, urethra, and anus are important pelvic structures crucial to define the best treatment option for the patient. Moreover, lymph nodes evaluation requests the integration of some imaging techniques such as MRI, PET/CT, and ultrasound. Radiologists should be familiar with MR imaging anatomy, technique, vulvar tumor characteristics, and spreading. The MRI report should provide a complete evaluation of the tumor in order to correctly stage LAVC and to give the clinicians all the necessary information. 

## Figures and Tables

**Table 1:** FIGO staging of vulvar carcinoma.

Figo Stage	Tumor Spread Description
I	Tumor confined to the vulva:
IA	lesions ≤ 2 cm in size, confined to the vulva and/or perineum with stromal invasion ≤ 1 mm. No nodal metastasis.
IB	lesions > 2 cm in size or any size with stromal invasion > 1 mm confined to the vulva and/or perineum. No nodal metastasis.
II	Tumor of any size with extension to adjacent perineal structures (lower 1/3 urethra, lower 1/3 vagina, anus). No nodal metastasis.
III	Tumor of any size with or without extension to adjacent perineal structures (lower 1/3 urethra, lower 1/3 vagina, anus) with involvement of inguinofemoral lymph nodes:
IIIA	with 1 lymph node metastasis (≥5 mm) or 1–2 lymph node metastases (<5 mm).
IIIB	with 2 or more lymph node metastases (≥5 mm) or 3 or more lymph node metastases (<5 mm).
IIIC	with positive lymph nodes with extracapsular spread.
IV	Tumor with invasion of other regional (upper 2/3 urethra, upper 2/3 vagina) or distant structures:
IVA	tumor of any size with extension to any of the following: upper/proximal 2/3 urethra; upper/proximal 2/3 vagina; bladder mucosa; rectal mucosa or fixed to pelvic bone or fixed or ulcerated inguinofemoral lymph nodes.
IVB	distant metastasis including pelvic lymph nodes.

**Table 2:** Summary of most relevant publications regarding the role of MRI for staging vulvar cancer.

1st Author	Year	Patient Number	Study Type	Results
Nikolić [11]	2021		Guidelines—Vulvar cancer staging: guidelines of the European Society of Urogenital Radiology (ESUR)	Guidelines for staging vulvar cancer with expert MRI recommendations of patient preparation, protocol, and structured report
Lakhman [26]	2021		Guidelines—ACR Appropriateness Criteria. Staging and follow-up of vulvar cancer	Summary of the literature and guidelines for the use of imaging for staging and follow-up of vulvar cancer
Serrado [12]	2019		Review—State of the art in vulvar cancer imaging	Imaging (MRI, CT, PET/CT) of vulvar cancer
Shetty [5]	2017		Review—MR Imaging of vulvar and vaginal cancer	MRI of vulvar and vaginal cancer
Agarwal [27]	2017		Review—MR imaging of the female perineum. Clitoris, labia, and introitus	MRI of female perineum, normal anatomy, benign and malignant pathology
Miccò [2]	2015		Review—Imaging features of uncommon gynecologic cancers	Imaging (US, CT, MR, PET/CT) of uncommon gynecologic cancers
Kim [7]	2013		Review—Update on imaging of vulvar squamous cell carcinoma	Imaging (MRI, PET/CT) for staging and follow-up of vulvar cancer
Viswanathan [14]	2013		Review—Multimodality Imaging of Vulvar Cancer: Staging, Therapeutic Response, and Complications	Imaging (MRI, CT, PET/CT) of vulvar cancer
Hosseinzadeh [15]	2012		Review—Imaging of the Female Perineum in Adults	Imaging of female perineum, normal anatomy and pathology of vulva, vagina, urethra and anus
Kataoka [19]	2010	49	Retrospective—The accuracy of magnetic resonance imaging in staging of vulvar cancer: a retrospective multi-centre study	Staging accuracy unenhanced MRI 69.4% (*n* = 36). Adding CE-MRI raised staging accuracy from 75% to 85% (*n* = 20).
Alt [28]	2011		Review—Imaging of Female Pelvic Malignancies Regarding MRI, CT and PET/CT	Imaging (MRI, CT, PET/CT) of vulvar, vaginal and ovarian cancer
Griffin [1]	2008		Review—Magnetic Resonance Imaging of vaginal and vulval pathology	MRI of vaginal and vulvar benign and malignant pathology
Sohaib [10]	2003		Review—Imaging in vulval cancer	Imaging (US, CT, MRI, PET, lymphoscintigraphy for sentinel lymph node) of vulvar cancer
Chang [29]	2002		Review—Imaging of the vagina and vulva	Imaging (MRI, US, CT, radiograph, lymphangiography) of vaginal and vulvar benign and malignant pathology
Sohaib [30]	2002	22	Retrospective—MR Imaging of carcinoma of the vulva	Moderate MRI correlation with clinico-pathological staging of the primary tumor and high specificity for the detection of nodal involvement

**Table 3:** MR Imaging protocol for evaluation of patients with vulvar cancer.

Sequence	Plane	Objective
T1WI pelvis	Axial	Panoramic view (lymph nodes, uterus, adnexa, bone)
T2WI pelvis	Axial	Panoramic view (lymph nodes, uterus, adnexa, bone)
T2WI pelvis	Sagittal	Vulva and adjacent structures (urethra, anus, vagina)
T2WI (small FOV)	Axial oblique (perpendicular to the long axis of the urethra)	Vulva and adjacent structures (urethra, anus, vagina)
T2WI (small FOV)	Coronal oblique (parallel to the long axis of the urethra)	Vulva and adjacent structures (urethra, anus, vagina)
DWI (b 0–1000) (small FOV)	Axial oblique as T2WI (perpendicular to the long axis of the urethra)	Tumor detection and extension
T2WI abdomen	Axial	Lymph nodes and hydronephrosis
T1WI Multhiphase DCE 3D GRE pelvis	Axial oblique (perpendicular to the long axis of the urethra)	Tumor detection and extension, lymph nodes
T1WI DCE 3D GRE pelvis	Coronal oblique (parallel to the long axis of the urethra)	Tumor detection and extension, lymph nodes

Legend: WI: weighted imaging; DWI: diffusion weighted imaging; DCE: dynamic contrast enhanced; 3D: three-dimensional; GRE: gradient-recalled echo.

## Data Availability

Not applicable.
